# Clinical Management of an Odontogenic Infection With an Extraoral Sinus Tract in an 11-Year-Old Child: A Case Report

**DOI:** 10.7759/cureus.116032

**Published:** 2026-09-10

**Authors:** Haneen Hesham, Farah Najib, Aania Adil

**Affiliations:** 1 Department of Dentistry, Royal Medical Services, Bahrain Defense Force Hospital, Riffa, BHR; 2 Department of Pediatrics, Royal Medical Services, Bahrain Defense Force Hospital, Riffa, BHR

**Keywords:** antibiotic therapy, extraoral sinus, facial cellulitis, odontogenic infection, streptococcus pyogenes infection

## Abstract

Odontogenic infections may initially present as facial cellulitis and, when left untreated, can progress to chronic infection, abscess formation, and extraoral sinus tract development. Early diagnosis and definitive management are essential to prevent complications and recurrent infection. This case report describes an 11-year-old girl who presented to the Pediatric Dental Clinic at the Dental and Maxillofacial Center (DMFC) with recurrent swelling on the left side of the face that had been intermittently treated with antibiotics for nearly one year. Clinical examination revealed an extraoral sinus tract in the left submandibular region associated with a severely carious lower left first molar. Cone-beam computed tomography (CBCT) played an important role in establishing the odontogenic origin of the infection, demonstrating the extent of periapical pathology and buccal cortical bone destruction. Following diagnosis, definitive treatment was achieved through extraction of the affected tooth. The patient showed complete resolution of the facial swelling and extraoral sinus tract following extraction, with no further recurrence reported during follow-up. This case highlights the importance of recognizing the progression of odontogenic infection from an initial acute presentation to a chronic extraoral sinus tract in pediatric patients. It also emphasizes the diagnostic value of CBCT in complex or recurrent cases and the importance of timely definitive dental treatment rather than repeated antibiotic therapy alone.

## Introduction

Odontogenic infections are a common cause of facial swelling in pediatric patients and may progress to cellulitis or abscess formation if left untreated [1]. These infections can extend from the dental pulp to the surrounding alveolar bone and adjacent soft tissues, leading to significant facial swelling and discomfort. 

Chronic extra-oral sinus tract and abscesses are among the most frequently encountered skin and soft tissue infections in clinical practice [1,2]. An extra-oral sinus tract is characterized by diffuse inflammation of the skin and subcutaneous tissues, presenting with erythema, warmth, swelling, and tenderness. In contrast, abscesses typically appear as localized collections of pus that may require surgical drainage in addition to antimicrobial therapy.

Appropriate management depends on accurate diagnosis and identification of the underlying cause, as the differential diagnosis of a chronic extraoral draining lesion in a child is broad and includes an odontogenic cutaneous sinus tract, an infected epidermoid or dermoid cyst, chronic bacterial skin infection, osteomyelitis, infected lymph nodes, and congenital lesions with secondary infection [1,2]. An odontogenic sinus tract should be considered when the lesion is located in the mandibular or submandibular region, particularly when associated with a history of dental caries or a nonvital tooth [3,4]. Careful clinical examination, dental evaluation, and appropriate imaging are essential to distinguish an odontogenic origin from other cutaneous and infectious conditions and to ensure that definitive treatment targets the underlying cause.

Epidemiological studies have shown that odontogenic infections account for a considerable proportion of facial infections in children and may occasionally require hospitalization [5,6].

## Case presentation

An 11‑year‑old girl presented to the Pediatric Dental Clinic at the Dental and Maxillofacial Center, accompanied by her mother, after referral from a general practitioner due to persistent facial swelling. The patient's mother reported intermittent swelling on the left side of the face over the previous year, which had been managed multiple times with antibiotic therapy. The patient had no known systemic diseases and no history of drug allergies.

Clinical examination

Extra-oral examination revealed a soft, localized swelling on the left cheek in the submandibular region, with a visible extra-oral sinus tract. No active discharge was observed during examination, and there were no signs of trismus or temporomandibular joint involvement (Figure 1).

Intraoral examination showed severe caries and mobility affecting the lower left first molar (LL6). Oral hygiene was poor, and generalized gingivitis was present. The contralateral first molar also demonstrated significant carious destruction.

**Figure 1:**
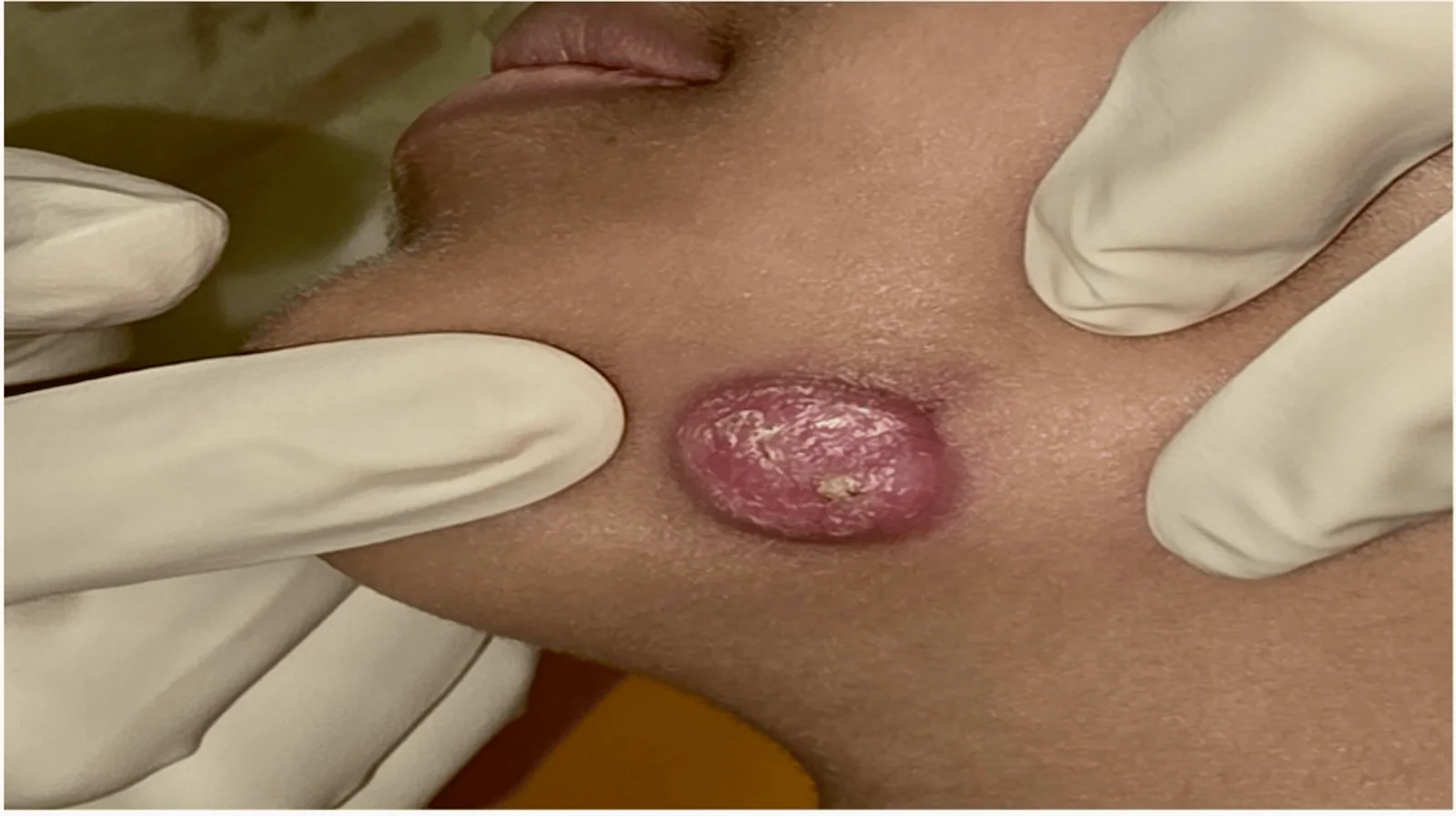
Extra-oral photograph showing localized swelling in the left submandibular area.

Cone-beam computed tomography (CBCT) imaging

Given the recurrent facial swelling, persistent extraoral draining sinus tract, and clinical suspicion of an underlying odontogenic infection, CBCT was performed further to evaluate the source and extent of the lesion. In this case, CBCT was considered appropriate because three-dimensional imaging was required to assess the extent of periapical pathology, evaluate the integrity of the buccal cortical plate, and clarify the relationship between the dental infection and the extraoral sinus tract. The extraoral sinus tract was identified clinically in the left submandibular region, and its course was assessed on CBCT without the insertion of a radiopaque material such as gutta-percha. CBCT revealed extensive destruction of the LL6 with discontinuity of the buccal cortical plate beneath the roots and a periapical radiolucent lesion consistent with chronic infection. The imaging findings demonstrated the sinus tract extending from the periapical region through the area of buccal cortical plate destruction toward the extraoral surface, supporting the odontogenic origin of the lesion (Figure 2).

**Figure 2:**
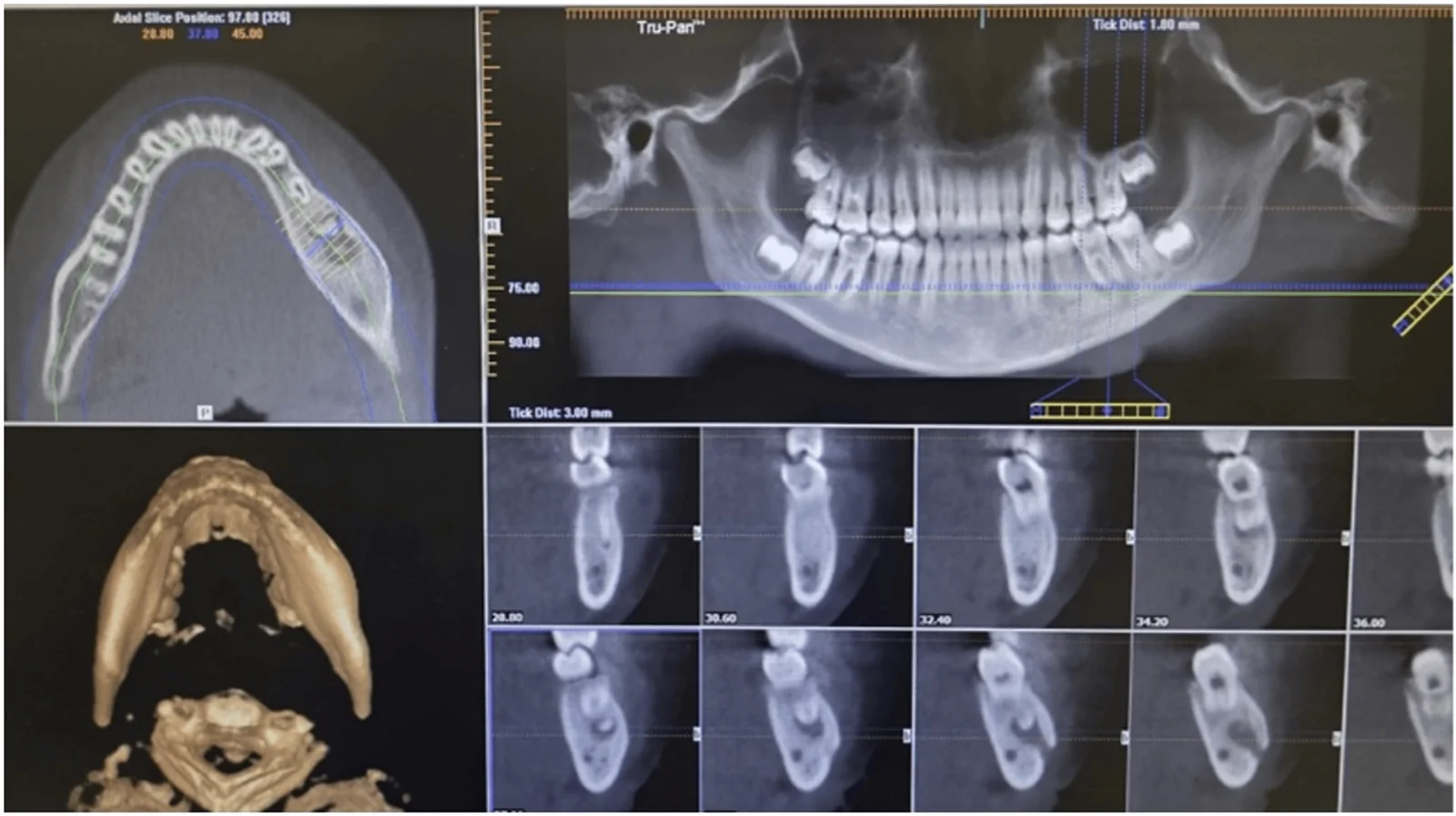
Preoperative cone-beam computed tomography (CBCT) demonstrating extensive periapical radiolucency associated with LL6 and buccal cortical plate perforation.

Initial management

Local anesthesia was achieved using 2% lignocaine with epinephrine (1:100,000) prior to the surgical extraction of LL6. Extraction was selected as the definitive treatment because the tooth exhibited extensive carious destruction with significant loss of tooth structure, associated chronic periapical infection, and discontinuity of the buccal cortical plate, resulting in a poor restorative and endodontic prognosis. Although endodontic treatment followed by definitive restoration may be considered for a restorable permanent first molar, the extent of structural destruction in this case made predictable restoration and long-term retention unfavorable. Therefore, extraction was considered the most appropriate approach to eliminate the source of infection and facilitate resolution of the associated extraoral sinus tract. The site was sutured with absorbable Vicryl sutures (3/0). Postoperative care instructions were provided, along with a prescription for the antibiotic amoxicillin 50 mg/kg twice daily for seven days and a referral to the plastic surgery department to evaluate the residual sinus tract and potential scar therapy.

Follow-up visits

*Post-operative Days Three to Five* 

Clinical observations showed a reduction in facial swelling, with all sutures remaining intact. The patient remained clinically stable, with no reported fever or other systemic signs of infection. Although there was no evidence of systemic involvement or active spreading infection at the time of extraction, antibiotics were prescribed as an adjunctive measure because of the chronic odontogenic infection, previous recurrent facial swelling, extraoral draining sinus tract, and extensive local infection associated with LL6. Definitive management was achieved by extracting the infected tooth, eliminating the underlying source of infection. The mother was reassured regarding the favorable progression of recovery, and the patient was advised to complete the prescribed antibiotic course.

One Week Follow-Up Appointment

Clinical examination demonstrated early scar formation at the site of the previous extraoral sinus tract, with a mildly raised and firm appearance. There were no clinical signs of superimposed infection or active inflammation. Given the patient's history and the appearance of the healing tissue, the possibility of abnormal scar formation was considered; however, a definitive diagnosis of keloid scar was not made at this early stage of healing. The patient was subsequently reviewed by the plastic surgery team, who recommended close observation and planned further scar assessment, with the possibility of future surgical intervention under general anesthesia (Figure 3).

**Figure 3:**
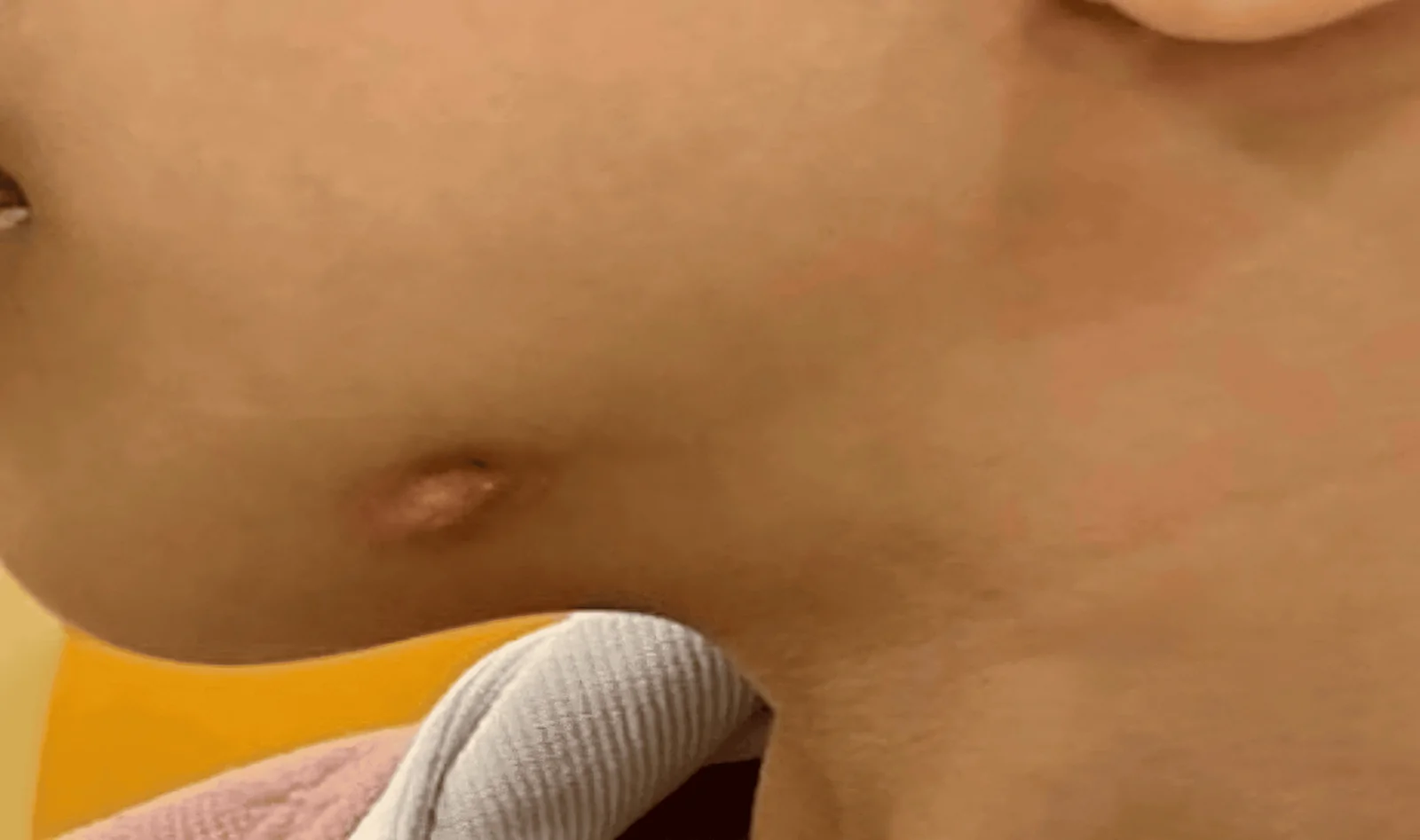
Extra-oral view demonstrating clinical improvement at one-week follow-up after management of odontogenic facial cellulitis.

Long-Term Follow-up

The patient remained clinically well, with complete resolution of the facial swelling and extraoral sinus tract and no evidence of recurrence of the odontogenic infection. The extraction site healed uneventfully, with no signs of infection or inflammation. The plastic surgery team monitored the scar at the previous sinus tract site, with continued assessment of its progression and appearance.

## Discussion

Odontogenic facial cellulitis is relatively common in children; however, the presence of chronic extraoral sinus tracts is less frequently reported [5]. Delayed diagnosis and repeated reliance on antibiotic therapy without definitive dental treatment may allow the infection to persist and progress.

Radiographic imaging, particularly cone-beam computed tomography (CBCT), plays an important role in identifying the extent of bone destruction and determining the pathway of odontogenic infection. Although conventional periapical radiography and panoramic radiography (orthopantomogram (OPG)) are useful first-line imaging modalities for assessing dental caries and periapical pathology. In this case, the recurrent facial swelling and persistent extraoral draining sinus tract warranted a more detailed assessment of the relationship among the affected tooth, the periapical lesion, the buccal cortical plate, and the extraoral soft tissues.

CBCT was therefore preferred as an adjunctive imaging modality because it provided multiplanar and three-dimensional visualization of the affected region. This allowed more accurate assessment of the extent of periapical bone destruction, the discontinuity of the buccal cortical plate, and the anatomical pathway of the infection toward the extraoral surface. Such information may not have been adequately demonstrated on a periapical radiograph or OPG because of anatomical superimposition. In addition, an OPG provides a broad overview of the jaws but has lower spatial resolution for detailed assessment of a localized periapical lesion, whereas a periapical radiograph provides greater detail of the tooth and periapical region but cannot reliably demonstrate the three-dimensional extent of cortical perforation or the spatial relationship of the lesion with adjacent structures. In this case, the additional anatomical information obtained from CBCT contributed to confirming the odontogenic origin of the extraoral sinus tract and assisted in definitive treatment planning [6].

However, the use of CBCT in a pediatric patient requires careful consideration of radiation exposure. Children are more sensitive to ionizing radiation than adults, and therefore the principles of justification and optimization are particularly important. CBCT should not be used routinely when conventional radiography can provide sufficient diagnostic information. In the present case, its use was justified by the atypical and recurrent clinical presentation, the persistent extraoral sinus tract, and the need to determine the extent and anatomical pathway of the underlying infection. The examination was undertaken when the expected diagnostic benefit was considered to outweigh the potential radiation risk, with the imaging field of view and exposure parameters selected to obtain the required diagnostic information while minimizing radiation dose. 

Management of odontogenic cellulitis often requires an interdisciplinary approach involving pediatric dentists, oral surgeons, and occasionally plastic surgeons. Early elimination of the infection source, combined with appropriate antimicrobial therapy, is essential for successful clinical outcomes [7].

## Conclusions

Odontogenic infections remain an important cause of facial cellulitis in pediatric patients and may occasionally present with extraoral sinus tracts. Early diagnosis, appropriate imaging, and prompt elimination of the infectious source are essential to prevent persistent infection and potential complications. Definitive management through timely elimination of the odontogenic source is critical for successful outcomes in odontogenic facial cellulitis. This case emphasizes the importance of early recognition of the dental origin, appropriate imaging, and prompt definitive dental treatment to achieve resolution and prevent recurrence. Antibiotics should be considered as an adjunct when clinically indicated, particularly in the presence of systemic involvement or spreading infection. Early multidisciplinary management may further support optimal recovery and appropriate management of residual soft-tissue changes.

## References

[1] Stevens DL, Bisno AL, Chambers HF (2014). Practice guidelines for the diagnosis and management of skin and soft tissue infections: 2014 update by the Infectious Diseases Society of America. Clin Infect Dis.

[2] Raff AB, Kroshinsky D (2016). Cellulitis: a review. JAMA.

[3] Biederman GR, Dodson TB (1994). Epidemiologic review of facial infections in hospitalized pediatric patients. J Oral Maxillofac Surg.

[4] Słotwińska-Pawlaczyk A, Orzechowska-Wylęgała B, Latusek K, Roszkowska AM (2023). Analysis of the clinical status and treatment of facial cellulitis of odontogenic origin in pediatric patients. Int J Environ Res Public Health.

[5] Giunta Crescente C, Soto de Facchin M, Acevedo Rodríguez AM (2018). Medical-dental considerations in the care of children with facial cellulitis of odontogenic origin. A disease of interest for pediatricians and pediatric dentists. Arch Argent Pediatr.

[6] Kuo J, Lin YT, Lin YTJ (2013). Odontogenic cellulitis in children requiring hospitalization. J Dent Sci.

[7] Uluibau IC, Jaunay T, Goss AN (2005). Severe odontogenic infections. Aust Dent J.

